# Designing double-site lipidated peptide amphiphiles as potent antimicrobial biomaterials to combat multidrug-resistant bacteria

**DOI:** 10.3389/fmicb.2022.1074359

**Published:** 2022-12-09

**Authors:** Zhenheng Lai, Hongyu Chen, Xiaojie Yuan, Jiahui Tian, Na Dong, Xingjun Feng, Anshan Shan

**Affiliations:** Laboratory of Molecular Nutrition and Immunity, Institute of Animal Nutrition, Northeast Agricultural University, Harbin, China

**Keywords:** antimicrobial peptides, peptide amphiphiles, double-site lipidated, multidrug-resistant bacteria, stability, mechanism of action

## Abstract

Rapidly evolving antimicrobial resistance and extremely slow development of new antibiotics have resulted in multidrug-resistant bacterial infections that present a serious threat to human health. Antimicrobial peptides (AMPs) provide promising substitutes, but more research is needed to address several of their present limitations, such as insufficient antimicrobial potency, high toxicity, and low stability. Here, we designed a series of novel double-site lipidated peptide amphiphiles based on a heptad repeat parent pentadecapeptide. The double-site lipidated peptide amphiphiles showed a broad spectrum of antimicrobial activities. Especially the double-site lipidated peptide amphiphile WL-C_6_ exhibited high potency to inhibit multidrug-resistant bacteria without significant toxicity toward mammalian cells. Furthermore, even at physiological salt ion concentrations, WL-C_6_ still exhibited outstanding antibacterial properties, and a sizeable fraction of it maintained its molecular integrity after being incubated with different proteases. Additionally, we captured the entire process of WL-C_6_ killing bacteria and showed that the rapid bacterial membrane disruption is the reason of bacterial death. Overall, WL-C_6_ shows great promise as a substitute for conventional antibiotics to combat the growing threat of multidrug-resistant bacterial infections.

## Introduction

Since the discovery of penicillin nearly a century ago, antibiotics have significantly contributed to the fight against pathogen infections and extended human life expectancy (Luepke et al., 2017). Antibiotics continue to be very important in the treatment and prevention of pathogenic illnesses today (Årdal et al., 2020). However, the growing issue of antibiotic resistance and the emergence of increasingly multidrug-resistant (MDR) bacteria have compelled scientists to look for novel treatments for pathogenic diseases (Laxminarayan et al., 2013). It is notable that the last line of defense for treating MDR gram-negative bacterial infections has been breached with the emergence and dissemination of *mcr* plasmid-mediated polymyxin resistance (Nang et al., 2019). Hence, there is an urgent need to develop novel antibiotics to combat MDR bacterial infections. Antimicrobial peptides (AMPs), due to their unique membrane disrupting mechanism of action, have emerged as one the promising alternatives to antibiotics (Ong et al., 2014). AMPs are small molecular active peptides and have been widely isolated and characterized from animals, plants, bacteria, fungi, protists, and archaea (Wang et al., 2016). Different AMPs show different biological functions, such as antibacterial (Shao et al., 2021; Panteleev et al., 2022; Wang et al., 2022), antibiofilm (Zai et al., 2021), antivirals (Ji et al., 2018), antifungal (Chou et al., 2021), anticancer and immunomodulatory (Lugo et al., 2019; Mookherjee et al., 2020). More than 22,400 AMPs have been isolated from nature or created synthetically in the lab as of this writing (Shi et al., 2022). Although these AMPs are diverse in sequence and structure, they typically have a high proportion of hydrophobic and positively charged amino acid residues (Wang et al., 2019). The positively charged residues promote the initial electrostatic interaction between the AMPs and bacteria. At the same time, hydrophobic residues facilitate AMPs’ insertion into the membrane following the initial electrostatic interactions. AMPs subsequently may disrupt the membrane through the barrel-stave, carpet, toroidal pore, or disordered toroidal pore, which causes fast cell death (Brogden, 2005; Hancock et al., 2021; Lai et al., 2022). Furthermore, various antibacterial modes of action that bypass the bacterial barrier and aim for intracellular sites to obstruct DNA replication, transcription, translation, protein synthesis or folding, among other processes, have been discovered (Graf et al., 2017; Schneider et al., 2017; Braffman et al., 2019; Hancock et al., 2021). In addition, many studies showed that AMPs or AMP polymers could kill bacteria *via* a multimodal mechanism (Lam et al., 2016; Lai et al., 2019, 2021). As a result, bacteria hardly ever develop AMP resistance.

Despite the fact that AMPs have shown a promising future in overcoming antibiotic resistance, several obstacles, such as insufficient bioactivities, high toxicity, susceptibility to protease degradation, and high production costs, continue to impede AMPs’ further development as commercially available drugs. Numerous strategies have been suggested to increase the antimicrobial potency and lessen the toxicity of AMPs, including template modification, minimalist *de novo* design, and design-based AMP libraries (Ong et al., 2014; Tan et al., 2020; Yang et al., 2020; Li et al., 2021; Wang C. et al., 2021; Wang Z. et al., 2021). Moreover, a variety of strategies have been investigated to increase the proteolytic stability of AMPs, including sequence changes, peptidomimetics, cyclization, N-methylation, PEGylation, lipidation, and glycosylation (Lai et al., 2022). Among others, peptide lipidation is a promising and attractive strategy for developing novel peptide-based antibiotics since it can be utilized to simultaneously improve the antimicrobial potency and the biostability of AMPs (Rounds and Straus, 2020).

Lipidation is the process of attaching one or more fatty acid chains to the lysine side chains or N-terminus of a peptide. Finding the ideal fatty acid chain length and the ideal modification sites is the primary objective of current research on the lipidation of AMPs (Rounds and Straus, 2020). The optimal chain lengths of lipidation for AMPs have been observed to most frequently fall between C_8_ and C_16_ in the literature (Laverty et al., 2010; Albada et al., 2012; Kamysz et al., 2020). We also observe that many researchers explicitly decide to modify AMPs or peptidomimetics with palmitic acid (Li et al., 2014a,b; Liu et al., 2020). Furthermore, the length of fatty acids that can enhance the bactericidal activity and selectivity of lipidated AMPs appears to be sequence dependent. For example, C_16_-G_3_(IIKK)_3_I-NH_2_, C_16_-(IIKK)_2_I-NH_2_, and C_16_-G_3_(IIKK)_2_I-NH_2_ were all conjugated with palmitic acid at the N-terminus, C_16_-G_3_(IIKK)_3_I-NH_2_ showed antimicrobial activity against *Streptococcus mutans*, while C_16_-(IIKK)_2_I-NH_2_ and C_16_-G_3_(IIKK)_2_I-NH_2_ did not kill the bacteria (Liu et al., 2020). For lipidated AMPs, there is often only one lipidated site, which is typically found at the N-terminus or C-terminus of the AMPs (Lei et al., 2018; Siriwardena et al., 2018; Gong et al., 2020). Double-site lipidation at the middle amino acid residues of the AMPs has rarely been explored. Therefore, it is imperative to research whether non-terminal double-site lipidation could be employed to improve the antibacterial properties and proteolytic stability of AMPs.

## Results and discussion

### Peptides design and synthesis

**Figure 1:**
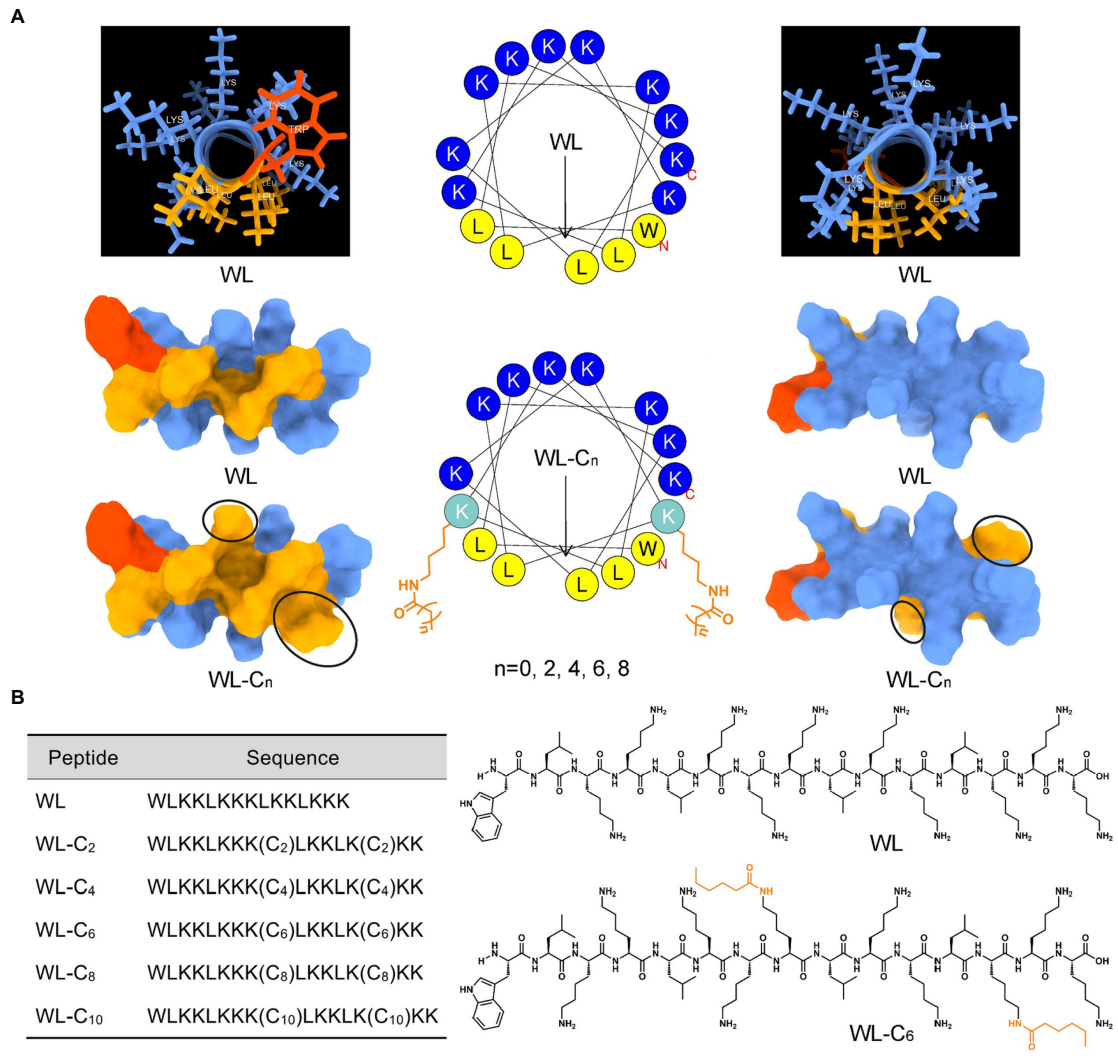
**(A)** Three-dimensional structure, helical wheel projections, and surface and ribbon views [hydrophobic faces (left) and hydrophilic faces (right)] of WL and WL-C_6_. **(B)** The sequences of WL and new lipidated peptides and the structural formula of WL and WL-C_6_ molecule.

Lipidation is a general and effective strategy to optimize the antimicrobial potency of AMPs (Lai et al., 2022). In the present study, we employ an α-helical structure pentadecapeptide (WL, WLKKLKKKLKKLKKK) as the parent peptide. Our lab created the pentadecapeptide by referencing a typical heptad repeat sequence (abcdefg). As shown in Figure 1A, the pentadecapeptide WL was rich in Lys, which providing multiple alternative lipidation attachment sites. More importantly, the pentadecapeptide WL had poor antibacterial activity, making it an excellent choice for researching how AMPs are affected by lipidation. In most cases, as the length of the fatty acid chain increases, the activity of the AMPs may increase at first and then decrease. Moreover, the length of the fatty acid cannot increase infinitely, and a clear positive correlation was observed between the length of the lipid moiety and cytotoxicity of the resulting compound (Niu et al., 2012). To minimize the toxic effect of lipidation, we used short fatty acids (C_2_ − C_10_) to modify the parent peptide WL simultaneously at two positions. The spatial separation of positive and hydrophobic residues (amphipathicity) may enhance the ability of AMPs to bind to bacterial membranes (Ong et al., 2014). Therefore, fatty acids were attached to the ε-amino of Lys^8^ and Lys^13^ to expect the perfect amphipathicity of the resulting compound (Figure 1A). The sequences of the novel lipidated peptides were presented in Figure 1B; Supplementary Figure S1. All the peptides used in the present study were synthesized by the SPPS method and preparative HPLC was employed to purify them all to a purity of >95%; the measured molecular weight was equal to the theoretical molecular weight, which indicated the peptides were successfully synthesized (Supplementary Figures S4–S15).

### Antibacterial properties and CD spectra

The antibacterial efficacy of the peptides was assessed by their measurement of minimum inhibitory concentrations (MICs; Table 1). The parent peptide WL exhibited poor activity toward all bacteria tested, with a geometric mean MIC of 64 μM. In the literature, different fatty acid length has been suggested as the optimum length to provide enhanced antimicrobial activity (Laverty et al., 2010; Albada et al., 2012). In one of our recent studies, we found the peptide dendron lipidated with palmitic acid (C_16_) was the most active peptide dendron (Lai et al., 2021). Despite the fact that the length of fatty acids that can enhance the bactericidal activity of lipidated antimicrobial peptides appears to depend on the peptide sequence (Liu et al., 2020), according to a very recent review, the optimal chain lengths are typically found to be between C_8_ and C_12_ (Rounds and Straus, 2020). However, in the present study, the MIC values of the lipidated AMPs clearly increased as the acyl chain length increased from C_2_ to C_6_. The lipidated peptide WL-C_6_ exhibited a minimum in the MICs, which showed a 3.43 μM in geometric mean MIC values. These results indicated that there might only need a shorter acyl chain length lipidated to achieve the minimum MICs for the double-site lipidated AMPs. Additional elongation of the acyl chain length had no further increase in antibacterial activity. What’s more, the lipidated peptide was almost completely inactivated in the tested concentrations when the acyl chain length increased to C_10_.

**Table 1:** Antibacterial activity of the peptides against a range of bacteria.

Peptides	MICs (μM)									
	*E. coli* 25922	*E. coli* UB1005	*P. aeruginosa* 27853	*S. typhimurium* 14028	*S. typhimurium* C7731	*S. aureus* 29213	*S. epidermidis* 12228	*E. faecalis* 29212	MRSA 43300	GM^a^
WL	32	32	32	32	32	128	128	>128	128	64.00
WL-C_2_	8	8	16	16	16	64	64	128	64	27.43
WL-C_4_	8	4	2	4	4	16	16	32	16	8.00
WL-C_6_	4	4	2	2	4	4	4	4	4	3.43
WL-C_8_	4	2	4	4	4	4	4	4	4	3.70
WL-C_10_	>64	16	>128	>128	>128	>128	>128	>128	>128	94.06

MIC is considered the lowest peptide concentration that no optical density increases. ^a^GM, geometric mean MICs from the 9 bacterial strains.

**Figure 2:**
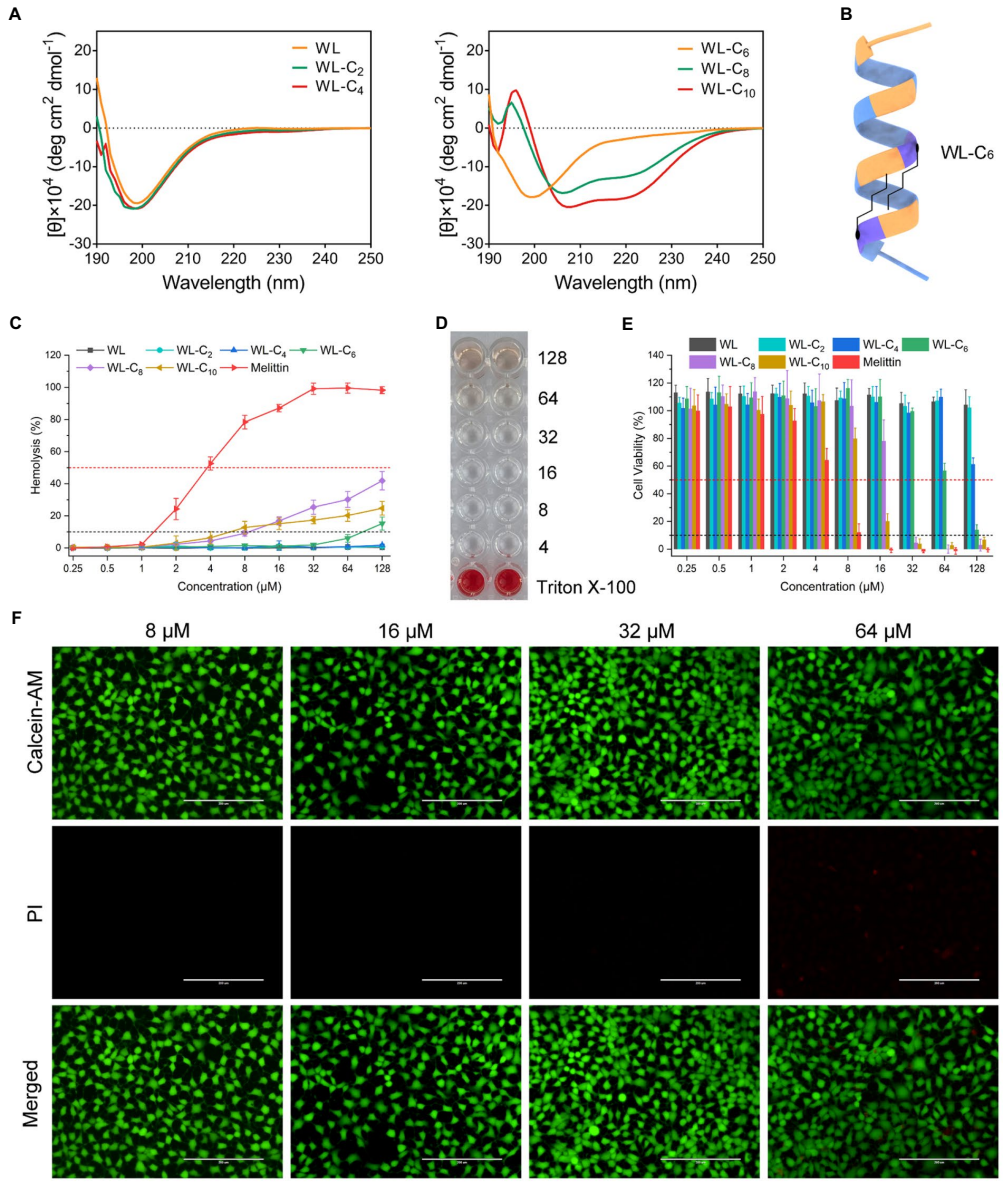
**(A)** CD spectra of the peptides in 10 mM PBS. **(B)** Model ribbon views of WL-C_6_. **(C)** Hemolytic activity of the peptides against hRBCs. Melittin was used as a control peptide. Data: mean ± standard deviation (SD; *n* = 6). **(D)** Hemolytic activity of WL-C_6_ (4–128 μM) against hRBCs. Erythrocyte suspension containing 0.1% TritonX-100 were used as a positive control. Data: mean ± SD (*n* = 6). **(E)** Cytotoxicity of the peptides against HEK293T. Melittin was used as a control peptide. **(F)** Live/dead cell staining images of HEK 293 cells after treating with WL-C_6_ and staining with PI (red) and Calcein-AM (green). Scale bar: 200 μm.

The change in the secondary structure may be responsible for the decreased antibacterial activities of the lipidated AMPs. As shown in Supplementary Figure S2, all peptides showed the characteristics of α-helical conformation in 30 mM SDS and 50% TFE, which exhibited a negative minimum of molar residue ellipticity at around 208 and 220 nm. However, with the acyl chain length increased, the secondary structure of the peptides converted from random coils into α-helices in PBS, which may be partly related to the gradually improved hydrophobic interaction of the two fatty acid chains (Figures 2A,B). The α-helices in the aqueous environment may constrain flexible movement at the membrane interface. The structure flexibility is necessary to enable the peptide to adopt different structural forms when interacting with the membrane for most conventional membrane-active AMPs (Gennaro and Zanetti, 2000; Hancock and Sahl, 2006). The lipidated peptides WL-C_8_ and WL-C_10_ all showed α-helical conformation in an aqueous environment. The formation of α-helical conformation in an aqueous environment may block the insertion of the peptides into the lipid bilayer, resulting in peptide inactivation.

Further studies utilizing multidrug-resistant *Escherichia coli* revealed that numerous antibiotics, including rifampicin, ceftazidime, erythromycin, and others, only weakly inhibited the growth of the multidrug-resistant *E. coli* (Table 2). Furthermore, the multidrug-resistant *E. coli* strains used in the current investigation were also colistin-resistant *E. coli* strains. They demonstrated resistance to polymyxin B and colistin, with MIC values for polymyxin B of 4 or 8 μM and colistin of 4 μM. In contrast, the lipidated peptides WL-C_6_ and WL-C_8_ displayed equivalent efficacy against *E. coli* HP73 and *E. coli* HP74, yielding MIC values of 4 μM, indicating that the lipidated peptide may be a potential antimicrobial agent to combat multidrug-resistant bacteria.

**Table 2:** Antimicrobial activity of the peptides against multidrug-resistant bacteria.

MICs (μM)		
	*E. coli* HP73	*E. coli* HP74
WL-C_6_	4	4
WL-C_8_	4	4
Polymyxin B	8	4
Colistin	4	4
Rifampicin	>128	16
Ceftazidime	128	32
Erythromycin	>128	>128
Gentamicin	128	128
Kanamycin A	>128	>128
Vancomycin	64	32
Ofloxacin	>128	>128
Norfloxacin	>128	128
Ciprofloxacin	>128	128

MIC is considered the lowest peptide concentration with no optical density increase.

### Biocompatibility of the lipidated peptides

We carried out hemolysis and cytotoxicity assays to assess the biocompatibility of the peptide in order to better understand its toxicity (Li et al., 2022b). WL, WL-C_2_, and WL-C_4_ (0.25–128 μM) exhibited no hemolytic activity after being exposed to fresh human erythrocytes (Figure 2C). Following treatment with 64 μM or less, WL-C_6_ displayed no detectable hemolytic activity; even after incubation at a very high concentration (128 μM, 32 × MIC), only ∼15% of red blood cells were burst (Figures 2C,D). The lipidated peptides WL-C_8_ and WL-C_10_ exhibited cytotoxic effects when the concentrations greater than 8 μM. However, erythrocytes occurred strong hemolysis after incubation with melittin, with hemolysis rate already greater than 50% at a melittin concentration of 4 μM. Furthermore, we investigated the cytotoxicity of the peptide toward human embryonic kidney cells (HEK293T and HEK293) and intestinal porcine enterocyte cells (IPEC-J2). WL, WL-C_2_, and WL-C_4_ exhibited no significant cytotoxicity at the test concentrations (Figure 2E; Supplementary Figure S3). WL-C_6_ displayed negligible toxicity toward HEK293T, HEK293, and IPEC-J2 cells when the peptide concentration was less than 64 μM (Figures 2E,F; Supplementary Figure S3). WL-C_8_ and WL-C_10_ demonstrated significant cytotoxicity toward HEK293T and IPEC-J2 cells at peptide concentrations greater than 32 μM. In contrast, melittin exhibited significant cytotoxicity to HEK293T or IPEC-J2 cells at concentrations greater than 8 or 16 μM. Based on careful consideration of antibacterial activity, hemolysis, and cytotoxicity, the lipidated peptide WL-C_6_ has the best antibacterial selectivity among the peptides listed in this study, which means that WL-C_6_ is more toxic to prokaryotic cells but has little effect on mammalian cells.

### Stability and bactericidal kinetics of the lipidated peptides

**Figure 3:**
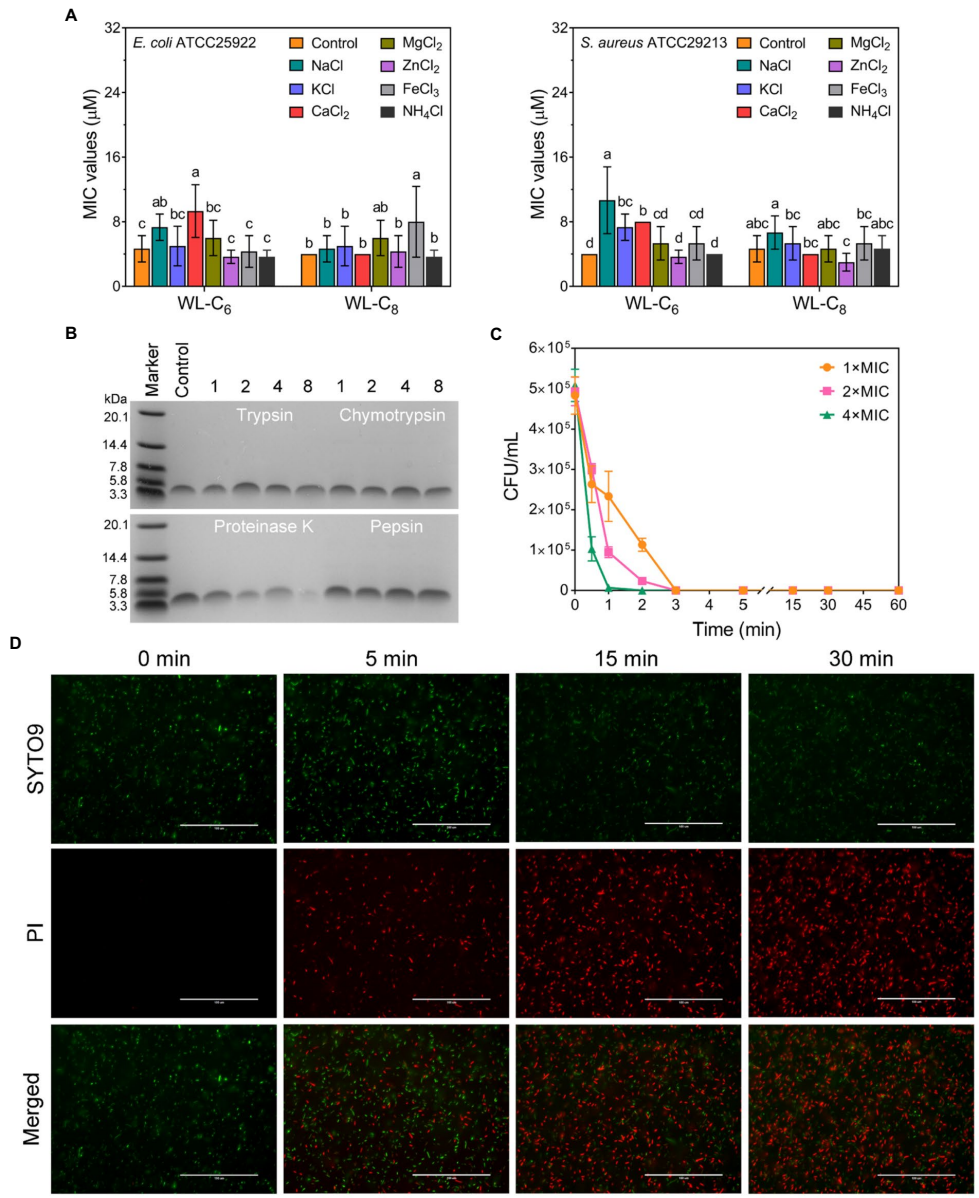
**(A)** Salt sensitivity of the lipidated peptides. To evaluate the peptide salt stability, the MICs of the lipidated peptides at physiological salt concentrations were employed. Data: mean ± SD (*n* = 6). The values with different superscripted letters (a–d) indicate a significant difference (*p* < 0.05). **(B)** 16.5% tricine−SDS − PAGE analysis of the WL-C_6_ after treatment with 500 μg/ml of chymotrypsin, trypsin, pepsin, or proteinase K for 1, 2, 4, and 8 h at 37°C. Control: untreated WL-C_6_. **(C)** Bactericidal kinetics of WL-C_6_ against *Escherichia coli* ATCC25922 at concentrations of 1 ×, 2 × and 4 × MIC. Data: mean ± SD (*n* = 3). **(D)** Fluorescence images of *E. coli* cells at points of time of 0-, 5-, 15-, and 30-min following treatment with 1 × MIC WL-C_6_ and stained with SYTO 9 (green) and PI (red). Scale bars, 100 μm.

The antimicrobial activity of AMPs is usually compromised by salts (Abou Alaiwa et al., 2014). To investigate the stability of the lipidated peptides, we tested the MICs of the lipidated peptides under physiological salt concentrations. As shown in Figure 3A, the MICs of WL-C_6_ under Na^+^, K^+^ and Ca^2+^ increased from 4 to 8 or 16 μM. The effects of NH_4_^+^, Zn^2+^, Mg^2+^, and Fe^3+^ on the antibacterial activity of WL-C_6_ against *E. coli* ATCC25922 and *S. aureus* ATCC29213 are negligibly significant. WL-C_8_ is more efficient in the presence of various salts, but its application is restricted by its toxicity toward mammalian cells. Proteases are widely found in plants, animal, and microorganisms (Song et al., 2022; Li et al., 2022a). Tryptase/chymase is widely distributed in human body tissues such the bronchus, skin, and cardiovascular system and has a functional structure that is comparable to that of trypsin/chymotrypsin (Irani and Schwartz, 1994; Miller and Pemberton, 2002). Therefore, to investigate the protease stability of the peptides, trypsin, chymotrypsin, and pepsin were utilized as the typical representatives of endogenous human proteases in the present study (Pan et al., 2020; Lai et al., 2022). We then employed 16.5% tricine−SDS − PAGE to analyze the residues of the lipidated peptides after they had been treated with 500 μg/ml of chymotrypsin, trypsin, pepsin, or proteinase K in order to investigate the protease resistance of WL-C_6_. As shown in Figure 3B, following incubation with 500 μg/ml of trypsin, chymotrypsin, or pepsin, the bands corresponding to WL-C_6_ were all still visible, proving that WL-C_6_ was not digested by these proteases. However, the bands corresponding to WL-C_6_ gradually vanished as the digestion period was longer than 1 h, suggesting that WL-C_6_ was cleaved by proteinase K (a kind of serine protease and used as the typical representatives of pathogen-secreted proteases in this paper). Notably, WL-C_6_ has extremely quick kinetics of killing, no *E. coli* cell survival occurred after adding 1 × MIC of WL-C_6_ for 3 min; and when the peptide concentration was up to 4 × MIC, the complete *E. coli* cell population died within 1 min (Figure 3C). These results indicated that the pathogen had been eliminated in a relatively brief period of time, but that this period was insufficient for the protease to digest WL-C_6_, implying that proteinase K has no impact on the peptide’s therapeutic benefits.

### Preliminary mechanistic studies

**Figure 4:**
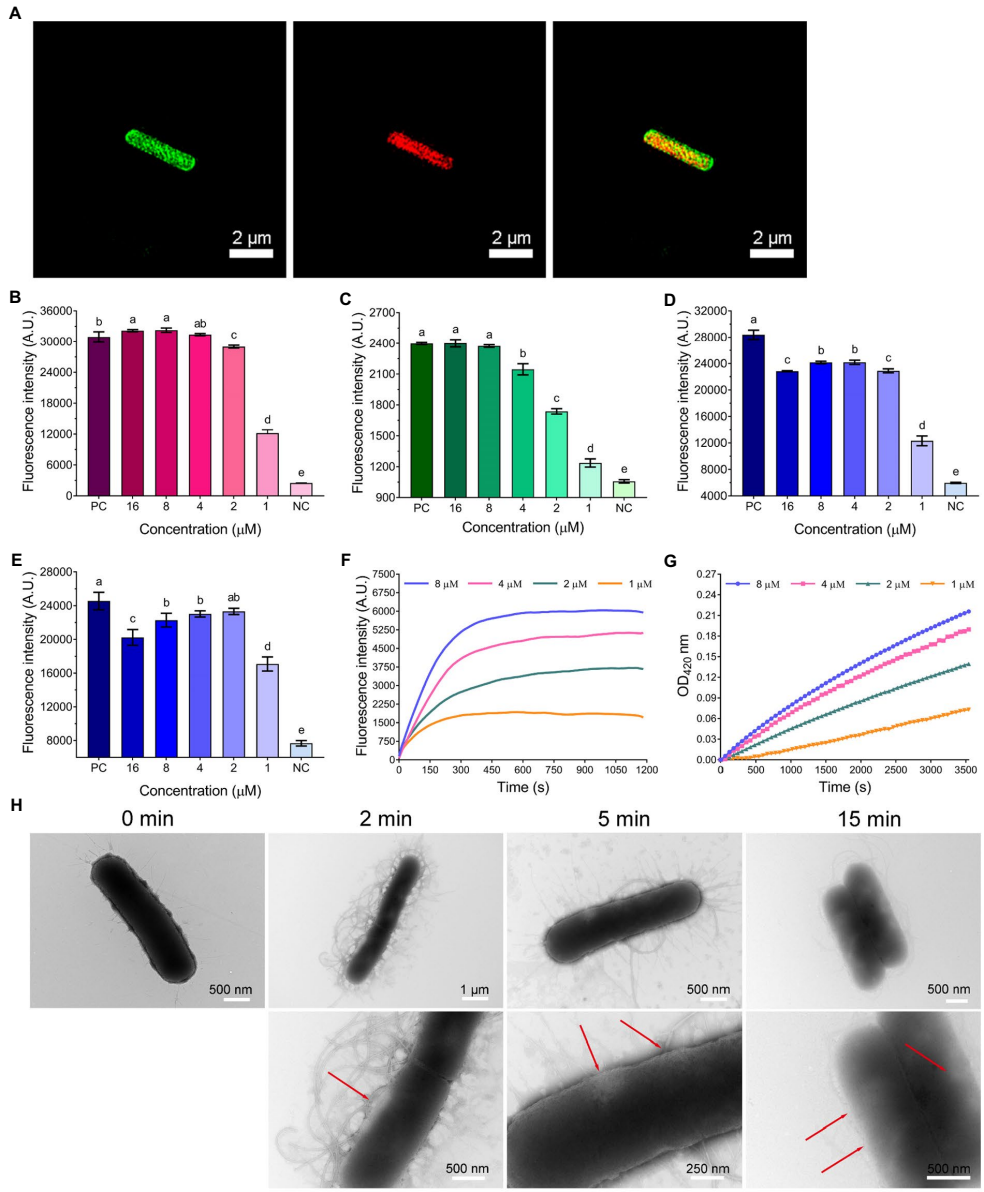
**(A)** 3D-SIM images of *E. coli* ATCC25922 following treatment with 1 × MIC FITC-labeled WL-C_6_. The green fluorescence signal is the FITC-labeled WL-C_6_, and the red fluorescence signal is the PI fluorescence signal. **(B)** LPS binding affinities of WL-C_6_. Positive control (PC): polymyxin B (20 μg/ml), negative control (NC): 1 × PBS. Data: mean ± SD (*n* = 3). **(C)** Outer membrane permeability induced by WL-C_6_. PC: polymyxin B (20 μg/ml), NC: 5 mM HEPES (pH = 7.4, containing 5 mM glucose). Data: mean ± SD (*n* = 3). **(D)** LUVs leakage induced by WL-C_6_, the ratio of POPC: POPG is 3:1. PC: Triton X-100 (0.1%), NC: 50 mM Tris–HCl buffer (pH = 7.4). Data: mean ± SD (*n* = 3). **(E)** LUVs leakage induced by WL-C_6_, the ratio of POPC: POPG is 1:3. PC: Triton X-100 (0.1%), NC: 50 mM Tris–HCl buffer (pH = 7.4). Data: mean ± SD (*n* = 3). **(F)** Cytoplasmic membrane depolarization induced by WL-C_6_. **(G)** Cytoplasmic membrane permeability induced by WL-C_6_. **(H)** Real-time negative staining TEM observation following WL-C_6_ treatment. The values with different superscripted letters (a–e) indicate a significant difference (*p* < 0.05).

As shown in Figure 3D, the bactericidal action of WL-C_6_ was rapid, and is similar to that observed for many AMPs, indicating that WL-C_6_ may adopt a similar membrane-disruptive mechanism of action (Song et al., 2019; Chou et al., 2021; Wang et al., 2022). Therefore, in order to gain a preliminary understanding of the WL-C_6_ action site, we conducted super-resolution fluorescence imaging using 3D structured illumination microscopy (3D-SIM) to monitor the localization site of WL-C_6_ in *E. coli*. As shown in Figure 4A, FITC-labeled WL-C_6_ peptides have attached to the *E. coli* cell surfaces, and at the same time, PI fluorescence (red) was also observed. This indicated that WL-C_6_ may exhibited antimicrobial activity against *E. coli* by acting on the cell membrane.

Therefore, we started by performing an lipopolysaccharides (LPS) binding experiment. The WL-C_6_ displayed a potent LPS binding ability when the peptide concentration was equivalent to or more than 2 μM (Figure 4B). It’s interesting to note that the MIC value of WL-C_6_ against *E. coli* is 4 μM, which indicates that WL-C_6_ interacts strongly with LPS on the outer membrane through electrostatic attraction even before reaching the MIC (Brogden, 2005; Lam et al., 2016). Because the potentially critical threshold concentration for membrane disruption has not yet been reached, *E. coli* has not been inhibited (Kepiro et al., 2020). To evaluate the WL-C_6_ induced membrane disruption, we first investigated the permeability of the outer membrane in response to the addition of various WL-C_6_ concentrations. As shown in Figure 4C, approximately 90% *E. coli* outer membrane disruption was found at 1 × MIC of WL-C_6_. Subsequently, to investigate whether WL-C_6_ may directly result in pore formation, we created large unilamellar vesicles (LUVs) with a 3:1 or 1:3 ratio of POPC:POPG. As shown in Figures 4D,E, the interaction of WL-C_6_ with these systems was dramatic. A significant increase in calcein fluorescence was observed after WL-C_6_ addition, which suggests that the WL-C_6_ induced membrane disruption may be the result of pore formation (Schnaider et al., 2017). Additionally, a rapid depolarization of the cytoplasmic membrane was observed following the addition of WL-C_6_, and the depolarization of the cytoplasmic membrane of *E. coli* nearly peaked after the addition of the peptides for 5 min (Figure 4F). Furthermore, a gradual increase in cytoplasmic membrane permeability was also detected (Figure 4G). These results indicate that the interaction between WL-C_6_ and the cytoplasmic membrane may induce cytoplasmic membrane depolarization, leading to membrane potential dissipation, which increased permeability and destroyed the integrity of the cytoplasmic membrane in *E. coli* (Lam et al., 2016).

**Figure 5:**
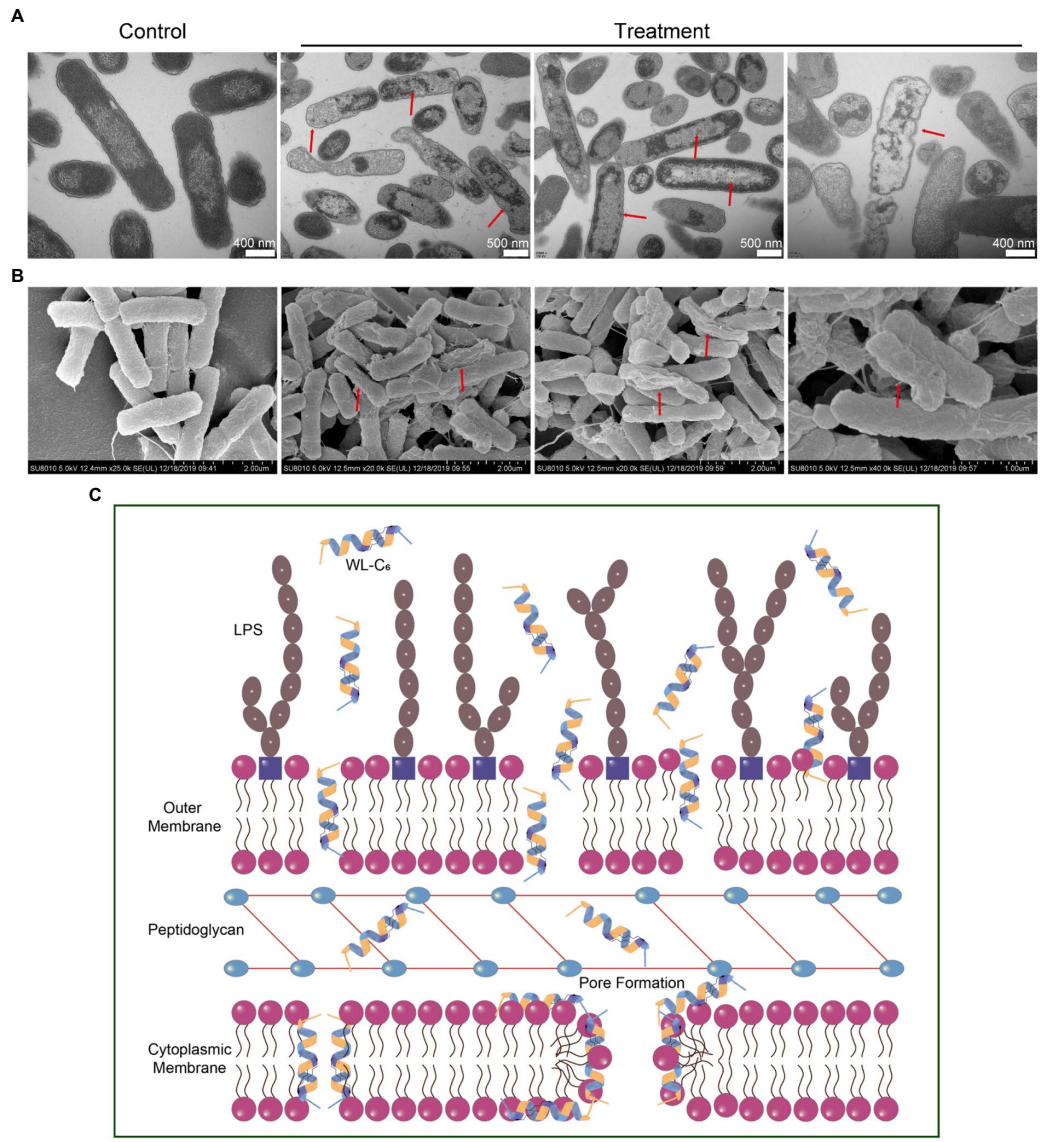
**(A)** TEM observations of morphological changes in *E. coli* cells following WL-C_6_ treatment for 1 h. **(B)** SEM observations of morphological changes in *E. coli* cells following WL-C_6_ treatment for 1 h. **(C)** The potential mechanism of action of WL-C_6_.

To gain a better insight into the mechanism of action through visual observation, we adopted transmission electron microscopy (TEM) and scanning electron microscopy (SEM) to visualize the morphological changes of *E. coli* cells after being treated with WL-C_6_. The *E. coli* cells without WL-C_6_ treatment were intact (Figure 4H, *t* = 0 min), the cytoplasm was homogeneous and full (Figure 5A, Control), and the cell surface was smooth (Figure 5B, Control). After 2 or 5 min of incubation, the bacterial membrane was partially ruptured, the intracellular milieu leaked out, the flagellum was broken and separated from the bacteria, and the cells were partially empty (Figure 4H, *t* = 2 or 5 min). Cavity and damaged *E. coli* cells were discovered at 15 min (Figure 4H). After incubating for 60 min (Figures 5A,B, Treatment), WL-C_6_ caused obvious outer membrane and cytoplasmic membrane separation, irregularly shaped holes in the membrane, and empty cells (Figure 5A, Treatment); SEM revealed that WL-C_6_ also caused bacterial shrinkage, break-off, and debris (Figure 5B, Treatment). These observations indicated that membrane disruption might be the main mechanism of action of WL-C_6_.

Taken together, based on the data presented above, we concluded that the WL-C_6_ initially adhered to the bacterial membrane through electrostatic interactions between the positively charged residues of WL-C_6_ and the LPS present on the bacterial membrane. The WL-C_6_ then inserted into the outer membrane and cytoplasmic membrane, causing damage to those membranes through pore formation or dissipation of transmembrane electrochemical ion gradients that allowed for the leakage of cellular contents and finally resulted to *E. coli* cell death (Figure 5C).

## Conclusion

In summary, our research has shown that double-site lipidation of the amino acid residues in the middle portion of the peptide can increase its antimicrobial activity without significantly increasing toxicity. Especially, the lipidated peptide WL-C_6_ showed excellent antibacterial potency against multidrug-resistant bacteria. The stability analysis demonstrated that the lipidated peptide WL-C_6_ could maintain effective antimicrobial activity in the presence of physiological concentrations of salt ions and was highly resistant to protease hydrolysis. Additionally, a series of fluorescence and microscopy experiments revealed that WL-C_6_ kills bacteria *via* a remarkable membrane damage mechanism. Overall, double-site lipidation was successfully exploited in the present study to produce peptide amphiphiles with high activity, high stability, and low toxicity while also successfully reducing the necessary fatty acid chain length. This work provides a promising alternative for the lipidated modification of AMPs and a new visual perspective for the development of antibacterial materials in biomedicine.

## Materials and methods

### Bacterial strains and peptide synthesis

*Escherichia coli* ATCC25922, *E. coli* UB1005, *Pseudomonas aeruginosa* ATCC27853, *Salmonella typhimurium* ATCC14028, *S. typhimurium* C7731, *Staphylococcus aureus* ATCC29213, Methicillin-resistant *S. aureus* (MRSA) ATCC43300, *S. epidermidis* ATCC12228, and *Enterococcus faecalis* ATCC29212 were stored by the institute of animal nutrition, Northeast Agricultural University (Harbin, China). Multidrug-Resistant *E. coli* HP73 and *E. coli* HP74 were clinically isolated from swine-derived *Escherichia coli* and stored by the institute of animal nutrition, Northeast Agricultural University (Harbin, China).

The peptides were synthesized by using a 9-fluorenylmethyloxycarbonyl (Fmoc)-based solid-phase peptide synthesis (SPPS) method. Then, the crude peptide was purified using high performance liquid chromatography (RP-HPLC). Subsequently, the molecular weights were confirmed using Electrospray Ionization Mass Spectrometry (ESI-MS, Linear Scientific Inc., United States).

### CD spectroscopy

A JASCO J-820 spectropolarimeter (Jasco, Japan) was used to collect CD spectra. CD spectra were collected using a 0.1 cm pathlength quartz cuvette at a scanning speed of 100 nm/min, 1 nm step, and 1 nm bandwidth. The ellipticities in mdeg were recorded after subtracting the solution background, and the output ellipticities data were the average of three scans. The molar ellipticity ([θ], deg. cm^2^ dmol^−1^) was calculated using the previously described equation (Lai et al., 2021). The experiment included two technical replicates.

### Measurement of minimum inhibitory concentrations

The MICs were determined using a broth microdilution method with reference to Clinical and Laboratory Standards Institute (CLSI) guidelines (Wiegand et al., 2008). After a two-fold serial dilution of the peptides (50 μl, dilution buffer: 0.01% acetic acid containing 0.2% BSA) prepared in a 96-well plate, a series of an equal volume of bacterial solution (50 μl) prepared in Mueller-Hinton broth (MHB) were added to the same 96-well plate, and the final bacterial inoculum size in each well of the 96-well plate was approximately 5 × 10^5^ CFU/ml. As positive and negative controls, bacteria solution without peptides and blank samples without bacteria were prepared in the same 96-well plate. The MIC was determined by optical density (OD) at 492 using a microplate reader (Tecan GENios F129004, Austria) after the plate was incubated at 37°C for 20 h. The lowest peptide concentration at which there was no increase in optical density was referred to as the MIC.

### Hemolysis assays

The hemolytic activity of the peptides was measured using fresh human erythrocytes as previously described (Zhu et al., 2020). The human blood was donated by three healthy donors, and they all acknowledged and consented to the use of their blood for the assay. The erythrocytes were obtained by centrifuging 1 ml of fresh human blood at 3,000 *g* for 3 min at 4°C. The erythrocytes were rinsed three times with 10 mM phosphate buffer saline (PBS, pH = 7.2) and resuspended in 10 ml of the same PBS to yield a 10% (vol/vol) suspension of human erythrocytes. Equal volumes of erythrocyte suspension (50 μl) and various concentrations of peptide solutions (50 μl, 256, 128, 64, 32, 16, 8, 4, 2, 1, 0.5, 0.25 μM) were incubated in a 96-well plate for 1 h at 37°C. Erythrocyte suspension without peptides and erythrocyte suspension containing 0.1% TritonX-100 were prepared in the same 96-well plate as negative and positive controls, respectively. The plate was centrifuged at 1,000 *g* for 10 min at 4°C. Aliquots (50 μl) of supernatant were transferred to a new 96-well plate and hemoglobin release was monitored at OD_570_ nm using a microplate reader (Tecan GENios F129004, Austria). Melittin was employed as a control peptide. Then the percentage of hemolysis was calculated using the previously described equation (Zhu et al., 2020).

### Cell cytotoxicity experiments

3-(4,5-dimethylthiozol-2-yl)-2,5-diphenyltetrazolium bromide (MTT) assay and live/dead cell staining assay were conducted to assess the peptide cytotoxicity. For the MTT assay, cell lines were seeded and passaged twice in high-glucose Dulbecco’s modified Eagle’s medium (DMEM, containing 100 μg/ml streptomycin, 100 U/ml penicillin, and 10% vol/vol fetal bovine serum) or Dulbecco’s Modified Eagle Medium/Nutrient Mixture F-12 (DMEM/F-12, containing 100 μg/ml streptomycin, 100 U/ml penicillin, and 10% vol/vol fetal bovine serum) at a 5% CO_2_ incubator (37°C). The cells (approximately 50,000 cells per well) were cultured in a 96-well plate at a 5% CO_2_ incubator for 1 day. Then various concentrations of peptide solutions (50 μl) were added to the plate and incubated for another 1 day. The cells solution without peptides and blank samples without cells and peptides were prepared in the same 96-well plate as positive and negative controls, respectively. Subsequently, MTT (0.5 mg/ml, 20 μl per well) was added and the plate was incubated for another 4 h. Removed all supernatant from the plate and added 150 μl of dimethyl sulfoxide. Finally, a microplate reader (Tecan GENios F129004, Austria) was used to record the absorbance data at OD_570_ nm. Melittin was employed as a control peptide. The cell viability was calculated using the previously described equation (Lai et al., 2021).

For live/dead cell staining assay, HEK293 cell lines, calcein-AM, and propidium iodide (PI) were employed to assess the cell viability. The processes that come next are the same as in the prior report (Lai et al., 2021). There were two technical replicates used in the experiment.

### Salt sensitivity assays

The salt stability of lipidated peptides was assessed by their antibacterial activity in the presence of physiological concentrations of salt ions. The experiment was conducted in the same way as previous MIC trials, except the BSA used in the experiment comprised either 300 mM NaCl, 9 mM KCl, 12 μM NH_4_Cl, 16 μM ZnCl_2_, 2 mM MgCl_2_, 4 mM CaCl_2_, or 6 μM FeCl_3_.

### Proteolytic stability assays

16.5% tricine−SDS − PAGE was used to evaluate the proteolytic stability of the peptides. The peptides (2.56 mM, 50 μl) were incubated with protease (500 μg/ml, 50 μl) at 37°C. After incubating for 1, 2, 4, or 8 h, the mixture (20 μl) was drawn out and placed in a bath of boiling water for 5 min. The mixture was then diluted to the suitable concentration for 16.5% tricine-SDS-PAGE analysis.

### Kill kinetics assays

*E. coli* ATCC 25922 cells were obtained using the same preparation method as the MIC assays. The *E. coli* cells (1 ml) at the concentration of 1 × 10^6^ CFU/ml were incubated with the peptides at the final concentrations of 1 × MIC, 2 × MIC, 4 × MIC. Aliquots were taken at *t* = 0, 0.5, 1, 2, 3, 5, 15, 30, and 60 min and diluted to a suitable concentration with 10 mM PBS (pH = 7.2). The diluent (20 μl) was spread on Mueller-Hinton Agar (MHA) plates and incubated overnight at 37°C. The numbers of the single colony were counted and expressed as CFU/ml. The live/dead bacteria staining assay is the same as described in our previous study (Lai et al., 2021).

### FITC-labeled peptide localization assays

*E. coli* ATCC25922 strains were cultured in MHB (10 ml) and incubated overnight at 37°C. The activated strain (100 μl) was transferred to a new MHB (10 ml). Bacteria cells were collected by centrifugation (5,000 g, 5 min) and washed three times with 10 mM PBS (pH = 7.2). The bacteria cells were resuspended in the same PBS at an OD_600_ of 0.1. A mixture of FITC-labeled WL-C_6_ and WL-C_6_ at a molar concentration ratio of 3:7 was then added to the *E. coli* solutions, resulting in a final concentration of 4 μM total peptides. After 30 min of incubation, PI solutions (0.1%, 1 mg/ml) were added to the mixtures. After another 10 min, the mixtures were centrifuged at 5,000 g for 5 min and washed 3 times with 10 mM PBS (pH 7.2). Following the fixation of 3 μl of the solution on a clean smear, the Deltavision OMX SR system (GE Healthcare, United States) with a 535 and 488 nm bandpass filter was used to observe the sample. The experiment was repeated twice.

### LPS binding assays

LPS (50 μg/ml) obtained from *E. coli* O111: B4 was incubated with the fluorescent probe BODIPY-TR-cadaverine (BC, 5 μg/ml) at 37°C for 4 h. The LPS-BC (50 μl/each well) mixture was added to a sterile 96-well plates, the peptide solutions were then added at various concentrations (50 μl). 50 μl of polymyxin B (20 μg/ml) or 1 × PBS was added as positive or negative control, respectively. The BC fluorescence data were collected using a microplate reader (Infinite 200 Pro, Tecan, China) at an excitation wavelength of 580 nm and an emission wavelength of 620 nm (Fang et al., 2021).

### Outer membrane permeability assays

After being transferred as one colony from the MHA plates, *E. coli* ATCC 25922 cells were grown aerobically to the mid-log phase. Then *E. coli* cells were collected by centrifuging them at 5,000 g for 5 min. *E. coli* cells were rinsed three times with 5 mM 4-(2-hydroxyethyl) piperazine-1-ethanesulfonic acid buffer (HEPES, pH = 7.4) containing 5 mM glucose and resuspended in the same buffer to yield an *E. coli* suspension with OD_600_ = 0.2. N-phenyl-1-naphthylamine (NPN), a fluorescent probe, was added to the *E. coli* suspension and incubated for 30 min at 37°C. The *E. coli*-NPN mixtures (100 μl) were incubated with various concentrations of peptide solutions (100 μl, diluted in HEPES with final concentration of 16, 8, 4, 2, and 1 μM). As a positive control, 100 μl of polymyxin B (20 μg/ml) was added to the *E. coli*-NPN mixtures (100 μl). As a negative control, 100 μl of 5 mM HEPES (pH = 7.4, containing 5 mM glucose) was added to the *E. coli*-NPN mixtures (100 μl). The NPN fluorescence data was collected immediately by a microplate reader (Infinite 200 Pro, Tecan, China) at an excitation wavelength of 350 nm and an emission wavelength of 420 nm.

### Cytoplasmic membrane permeability and depolarization assays

*o*-nitrophenyl-β-D-galactopyranoside (ONPG) degradation assays were used to determine cytoplasmic membrane permeability. The cytoplasmic membrane depolarization assays were performed with the membrane potential-sensitive fluorescent dye 3,3′-dipropylthiadicarbocyanine (diSC3-5). The testing procedure is the same as described in our previous reports (Wang Z. et al., 2021). We would not go into detail here in order to avoid repetition as much as possible.

### LUVs leakage assay

As we described previously, LUVs enveloped with 70 mM calcein were produced with 1-palmitoyl-2-oleoyl-sn-glycero-3-phosphocholine (POPC) and 1-palmitoyl-2-oleoyl-snglycero-3-phosphoglycerol (POPG) in the ratio of 3:1 or 1:3 (Shao et al., 2021). The LUVs were then incubated in a sterile 96-well plate with various concentrations of peptide solutions in 50 mM Tris–HCl buffer (pH = 7.4), with the final lipid concentration of LUVs equal to 100 μM and the final peptide concentration equal to 16, 8, 4, 2, or 1 μM. As a positive control, Triton X-100 (0.1%) was used. The plate was left in the dark for 30 min. Following that, the calcein fluorescence was measured using a microplate reader (Infinite 200 Pro, Tecan, China) at 490 nm excitation and 520 nm emission wavelengths.

### Real-time negative staining TEM observation

*E. coli* ATCC25922 cells were collected as we described in the outer membrane permeability assays and resuspended in 10 mM phosphate buffer (pH = 7.2) to yield an *E. coli* suspension with OD_600_ = 0.2. The following processing steps are then the same as previously described (Lai et al., 2021).

### SEM and TEM characteristics

*E. coli* ATCC 25922 cells were prepared in the same manner as described in the outer membrane permeability assays. Then the peptides (final concentration = 1 × MIC) were added to the *E. coli* cells (OD_600_ = 0.4) and incubated at 37°C for 60 min. Centrifugation at 5,000 *g* for 5 min was used to collect *E. coli* cells. The bacteria morphology was then fixed with glutaraldehyde (2.5% w/v). The following processing steps are identical to previously reported (Chou et al., 2020).

### Statistical analysis

Unless otherwise stated, all experiments were repeated independently at least three times with two technical replicates. All data were presented as the mean ± standard deviation (SD). Statistical analysis was carried out using SPSS 25.0 and a one-way ANOVA with Duncan’s multiple range test, where differences were regarded as statistically significant with probability *p* < 0.05.

## Data availability statement

The raw data supporting the conclusions of this article will be made available by the authors, without undue reservation.

## Ethics statement

The human blood samples used in this study were reviewed and approved by the Ethics Committee of the Northeast Agricultural University Hospital, Harbin, China (NEAUEC20200215).

## Author contributions

ZL and AS designed and conceived this work. ZL, HC, XY, and JT conducted the main experiments assay. ZL wrote the main manuscript text. AS, ND, and XF supervised the work and revised the final version of the manuscript. All authors have read and approved the final version of the manuscript.

## Funding

This work was supported by the National Natural Science Foundation of China (32030101, 31872368, and U21A20252), and the Natural Science Foundation of Heilongjiang Province (TD2019C001).

## Conflict of interest

The authors declare that the research was conducted in the absence of any commercial or financial relationships that could be construed as a potential conflict of interest.

## Publisher’s note

All claims expressed in this article are solely those of the authors and do not necessarily represent those of their affiliated organizations, or those of the publisher, the editors and the reviewers. Any product that may be evaluated in this article, or claim that may be made by its manufacturer, is not guaranteed or endorsed by the publisher.
