# Supplementary material for: Designing double-site lipidated peptide amphiphiles as potent antimicrobial biomaterials to combat multidrug-resistant bacteria
Source: Front Microbiol. 2022 Dec 9;13:1074359. doi: 10.3389/fmicb.2022.1074359 (PMC9780499; doi:10.3389/fmicb.2022.1074359)
Supplement: Supplementary file 1 [file Data_Sheet_1.docx]

**Supplementary Material**

**Designing double-site lipidated peptide amphiphiles as potent antimicrobial biomaterials to combat multidrug-resistant bacteria**

Zhenheng Lai, Hongyu Chen, Xiaojie Yuan, Jiahui Tian, Na Dong, Xingjun Feng, Anshan Shan*

Institute of Animal Nutrition, Northeast Agricultural University, Harbin 150030, P. R. China

* E-mail: [asshan@neau.edu.cn](mailto:asshan@neau.edu.cn). Orcid.org/0000-0003-2830-7509

**Supplementary Figures**


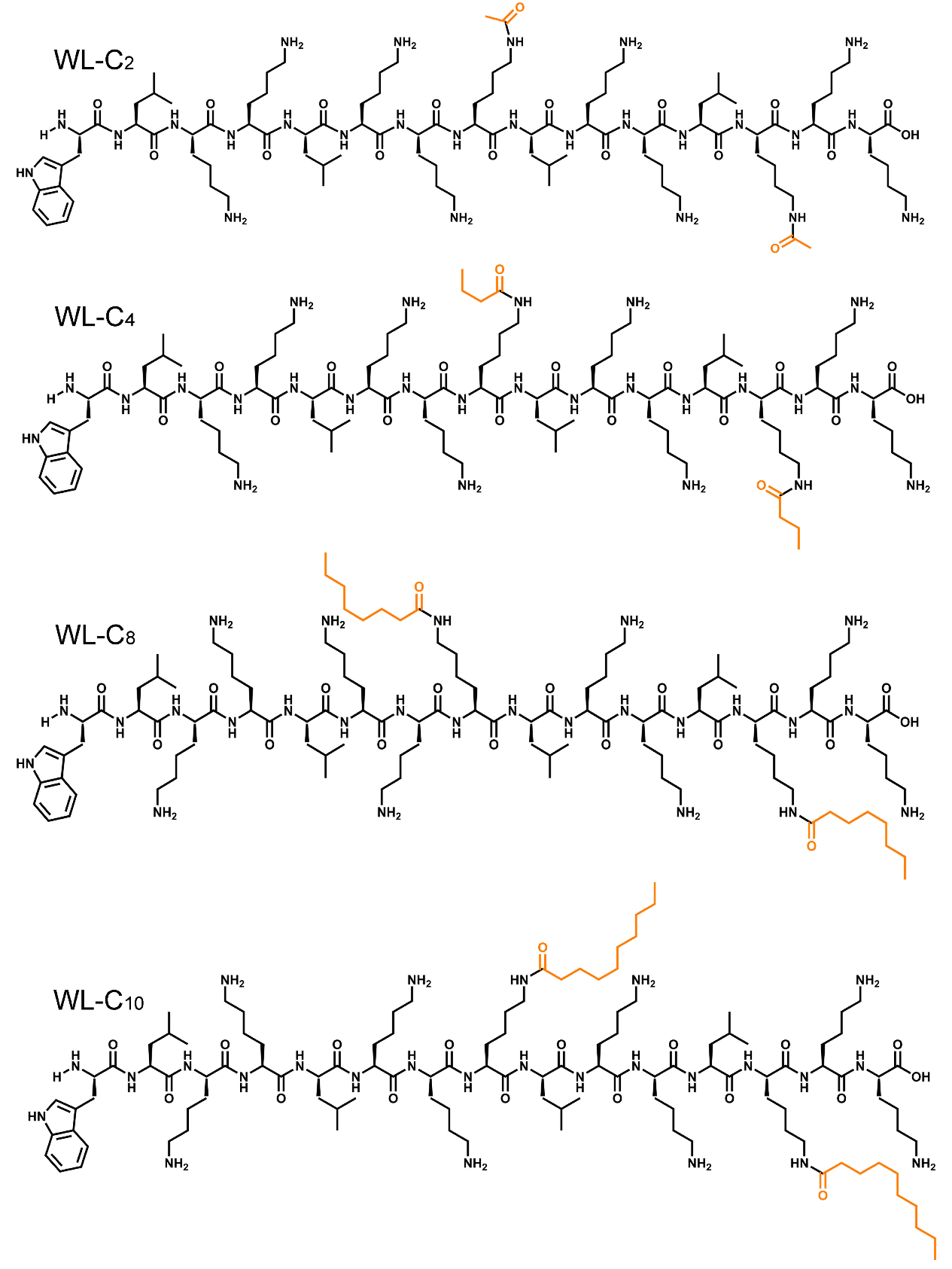


**Figure S1**. Structure of the lipidated peptides.


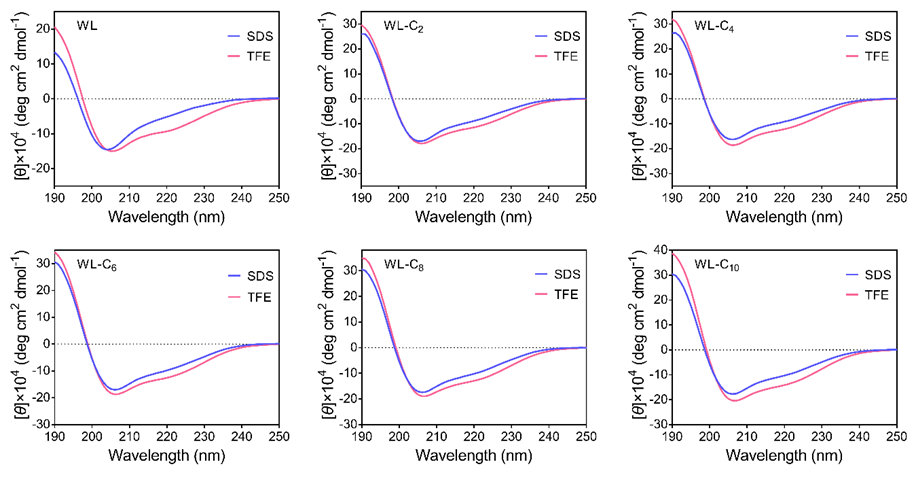


**Figure S2.** CD spectra of the peptides in 30 mM SDS (mimicking negatively charged prokaryotic membrane environments) and 50% TFE (mimicking a hydrophobic environment).


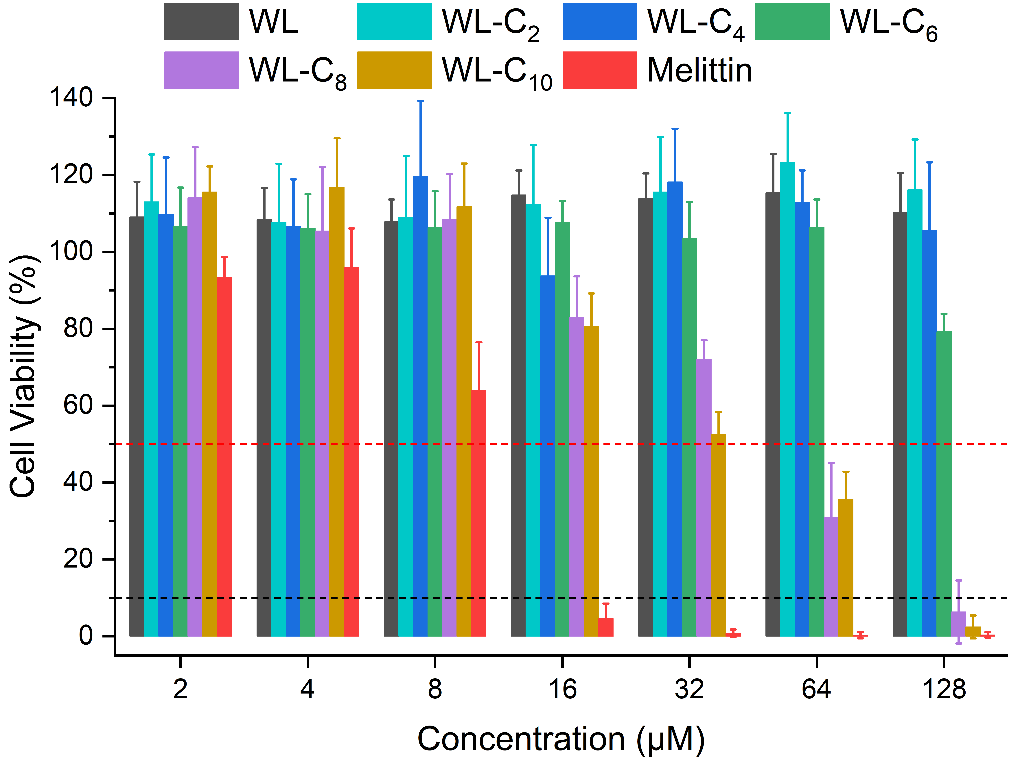


**Figure S3.** Cytotoxicity of the peptides against intestinal porcine enterocyte cells (IPEC-J2). Melittin was used as a control peptide.


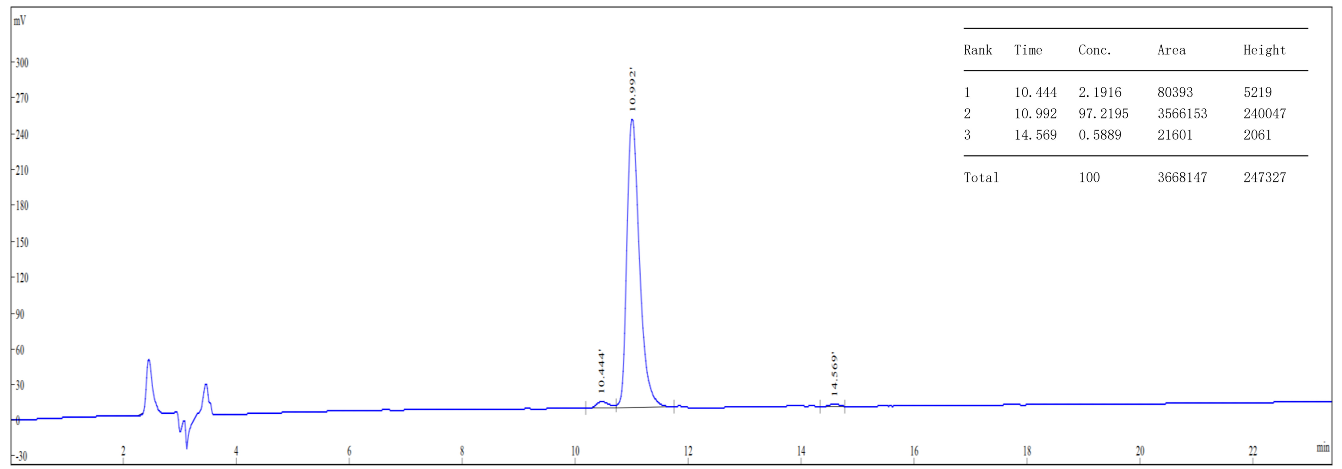


**Figure S4.** Reversed-phase high-performance liquid chromatography (RP-HPLC) chromatograms of WL. Gradient: 0.01 min: 90% A, 10% B; 25 min: 65% A, 35% B; 20.01 min: 0% A, 100% B; 30 min: stop (A: 0.1% trifluoroacetic in water, B: 0.1% trifluoroacetic in acetonitrile).


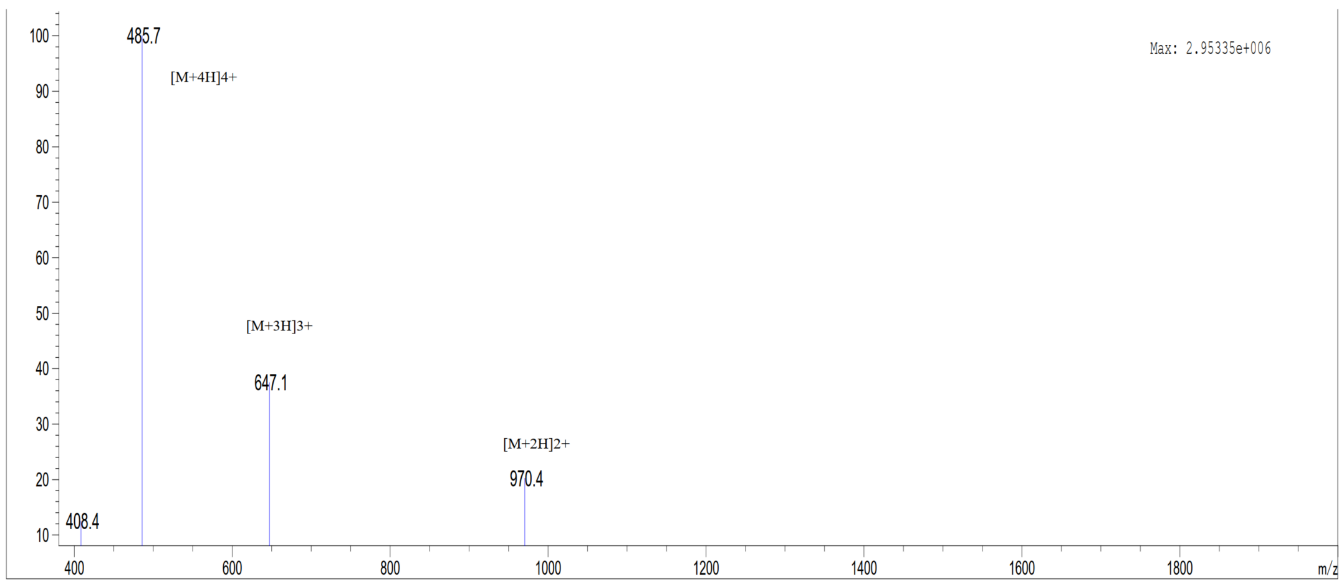


**Figure S5.** Electrospray Ionization Mass Spectrometry (ESI-MS) of WL. Expected mass: 1938.62; Measured average mass 1938.57.


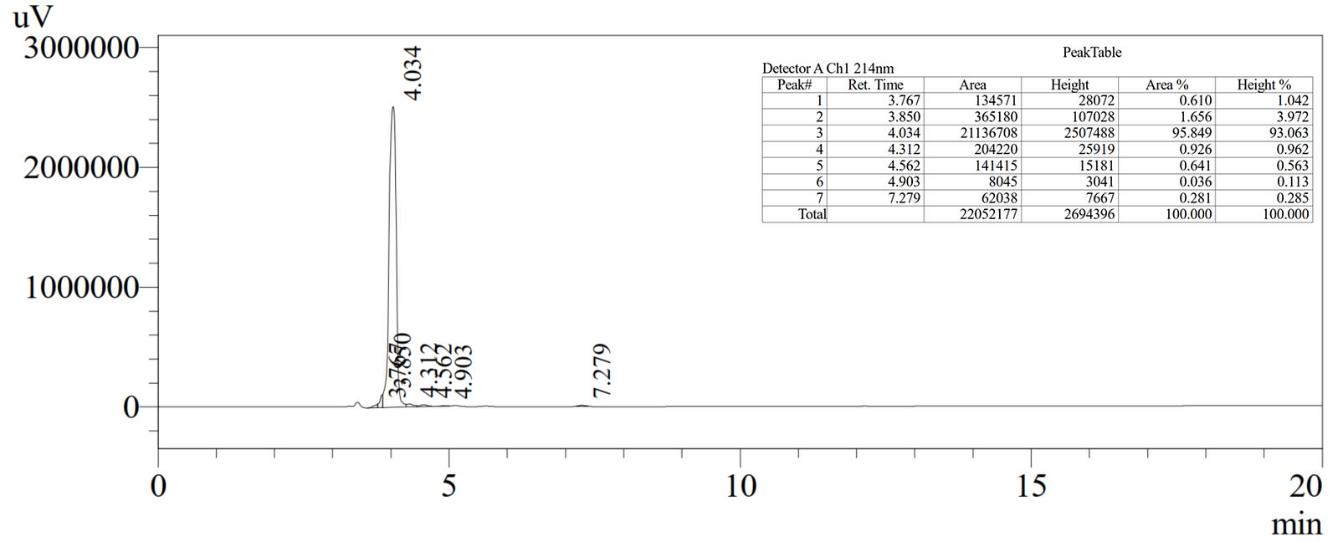


**Figure S6.** RP-HPLC chromatograms of WL-C_2_. Gradient: 0.01 min: 83% A, 17% B; 20 min: 63% A, 37% B; 20.01 min: 5% A, 95% B; 27.01 min: 5% A, 95% B; 27.02 min: stop (A: 0.1% trifluoroacetic in water, B: 0.1% trifluoroacetic in acetonitrile).


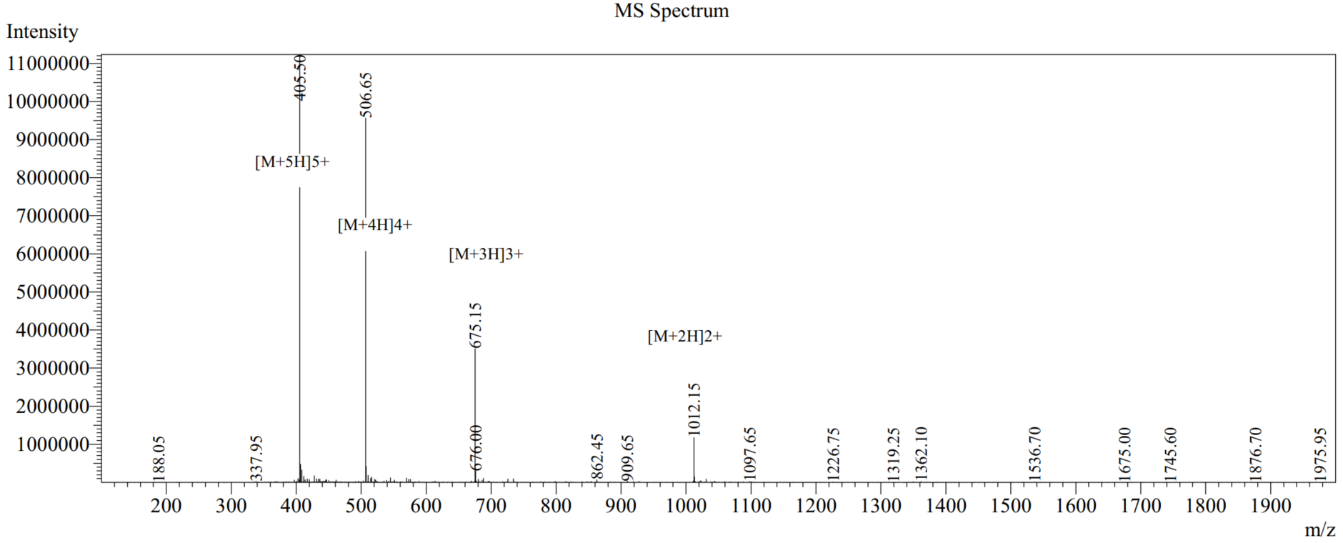


**Figure S7.** ESI-MS of WL-C_2_. Expected mass: 2022.69; Measured average mass 2022.50.


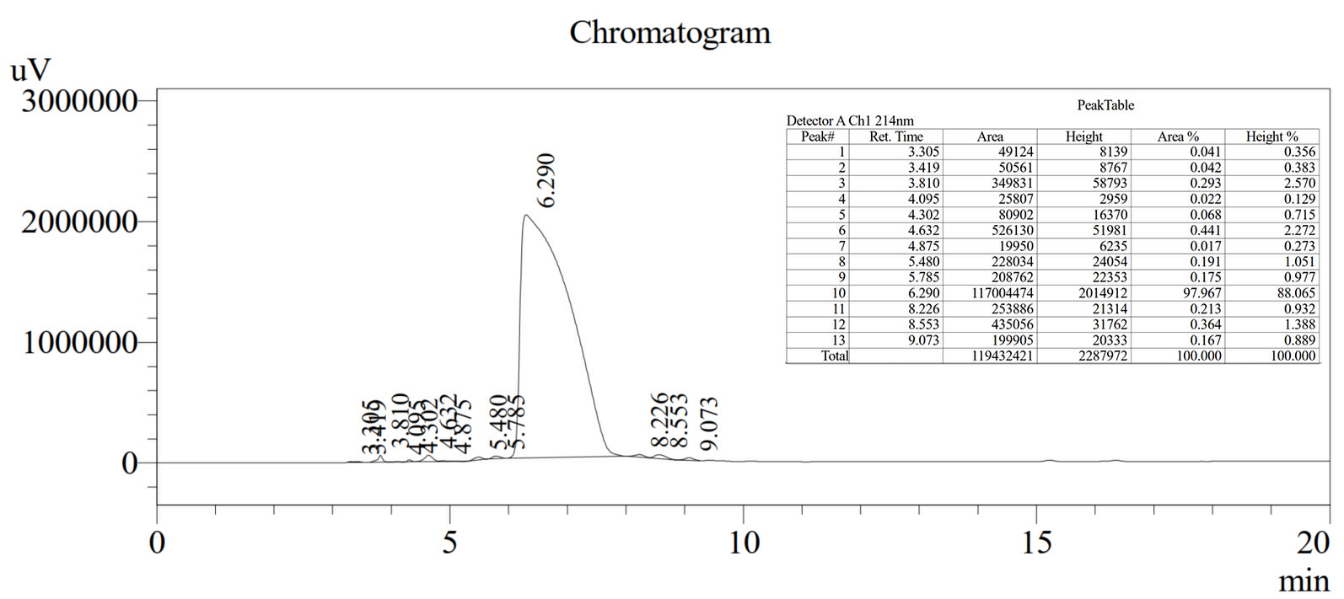


**Figure S8.** RP-HPLC chromatograms of WL-C_4_. Gradient: 0.01 min: 82% A, 18% B; 20 min: 62% A, 38% B; 20.01 min: 5% A, 95% B; 27.01 min: 5% A, 95% B; 27.02 min: stop (A: 0.1% trifluoroacetic in water, B: 0.1% trifluoroacetic in acetonitrile).


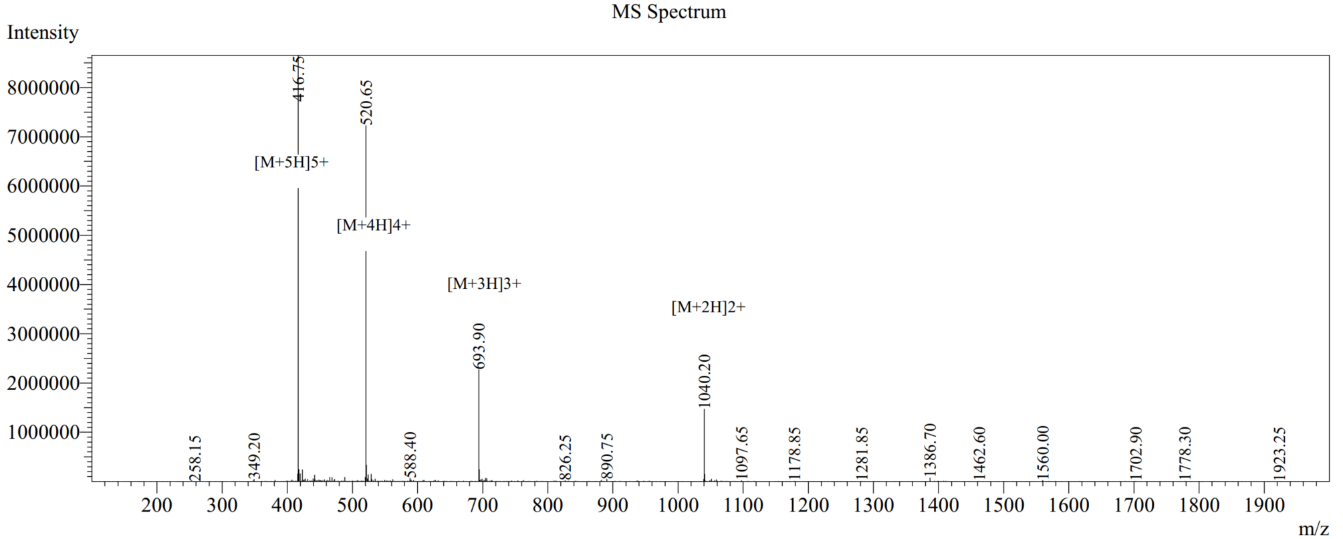


**Figure S9.** ESI-MS of WL-C_4_. Expected mass: 2078.80; Measured average mass 2078.75.


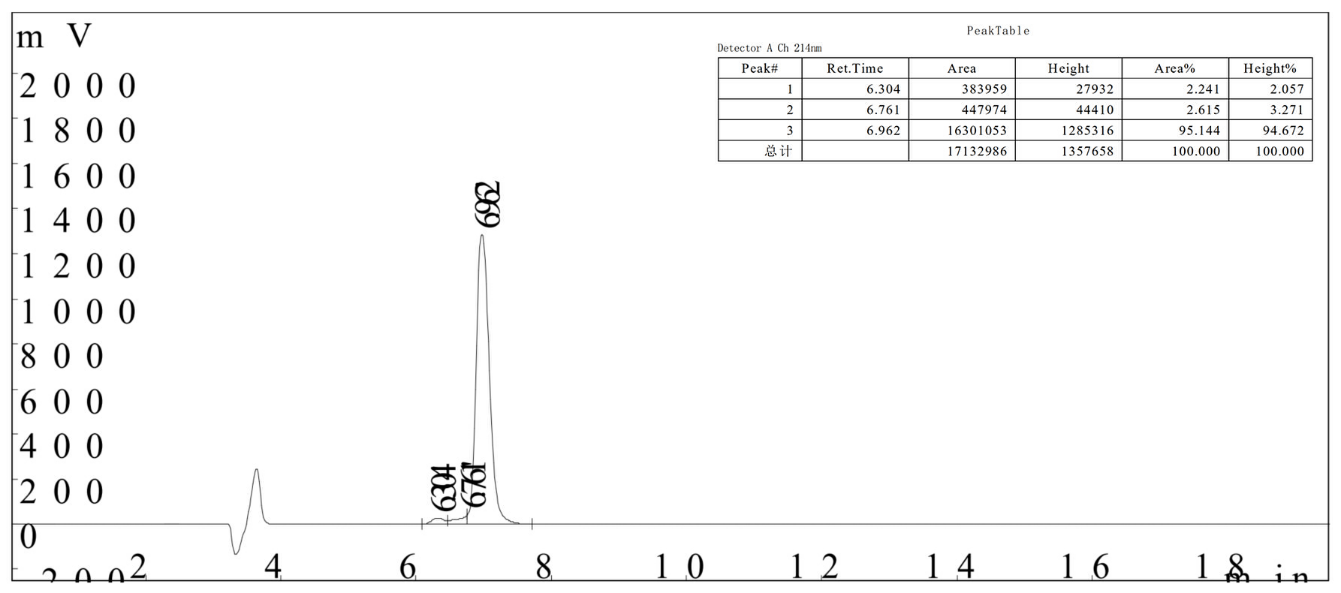


**Figure S10.** RP-HPLC chromatograms of WL-C_6_. Gradient: 0.01 min: 81% A, 19% B; 20 min: 61% A, 39% B; 28 min: 5% A, 95% B; 32 min: 5% A, 95% B; 35 min: stop (A: 0.1% trifluoroacetic in water, B: 0.1% trifluoroacetic in acetonitrile).


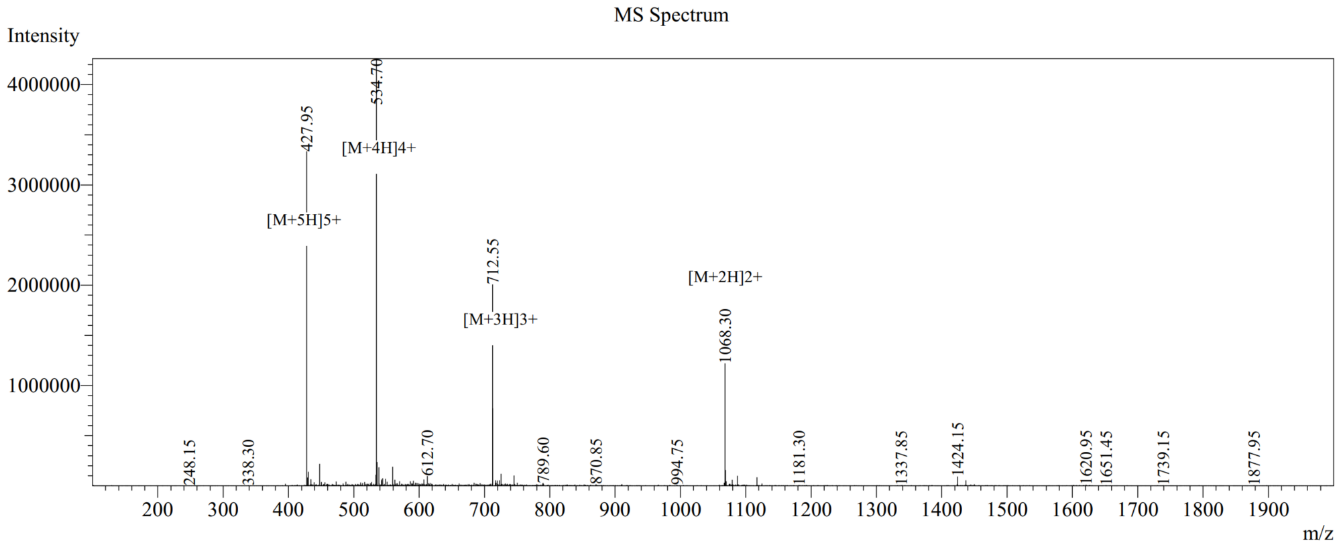


**Figure S11.** ESI-MS of WL-C_6_. Expected mass: 2134.91; Measured average mass 2134.80.


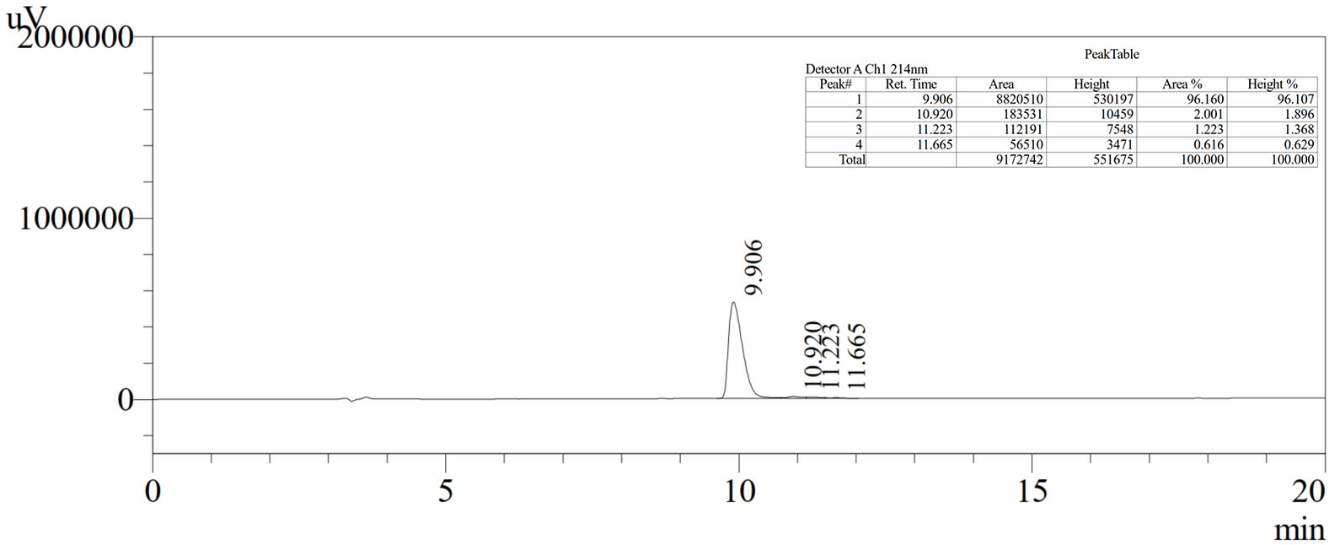


**Figure S12.** RP-HPLC chromatograms of WL-C_8_. Gradient: 0.01 min: 73% A, 27% B; 20 min: 53% A, 47% B; 20.01 min: 5% A, 95% B; 27.01 min: 5% A, 95% B; 27.02 min: stop (A: 0.1% trifluoroacetic in water, B: 0.1% trifluoroacetic in acetonitrile).


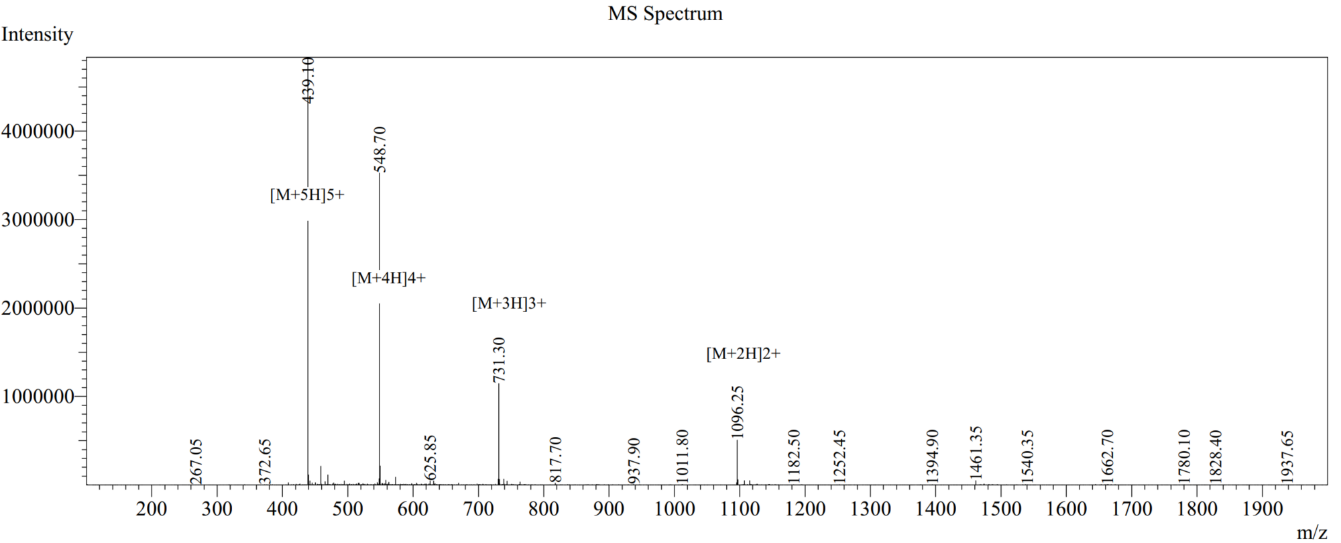


**Figure S13.** ESI-MS of WL-C_8_. Expected mass: 2191.02; Measured average mass 2190.50.


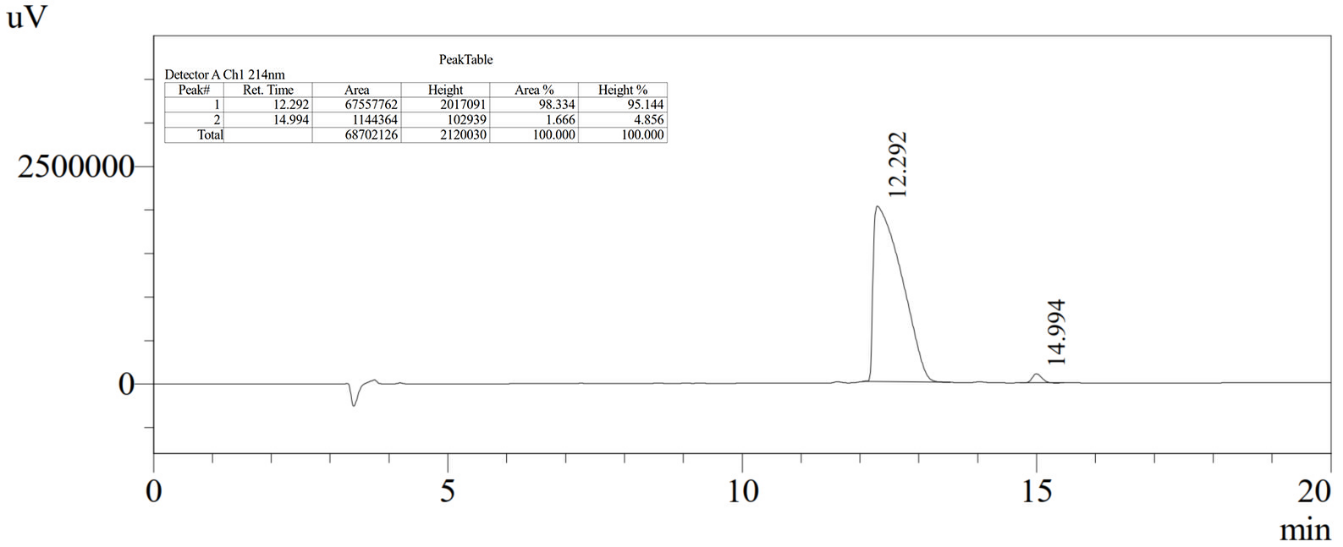


**Figure S14.** RP-HPLC chromatograms of WL-C_10_. Gradient: 0.01 min: 73% A, 27% B; 20 min: 53% A, 47% B; 20.01 min: 5% A, 95% B; 27.01 min: 5% A, 95% B; 27.02 min: stop (A: 0.1% trifluoroacetic in water, B: 0.1% trifluoroacetic in acetonitrile).


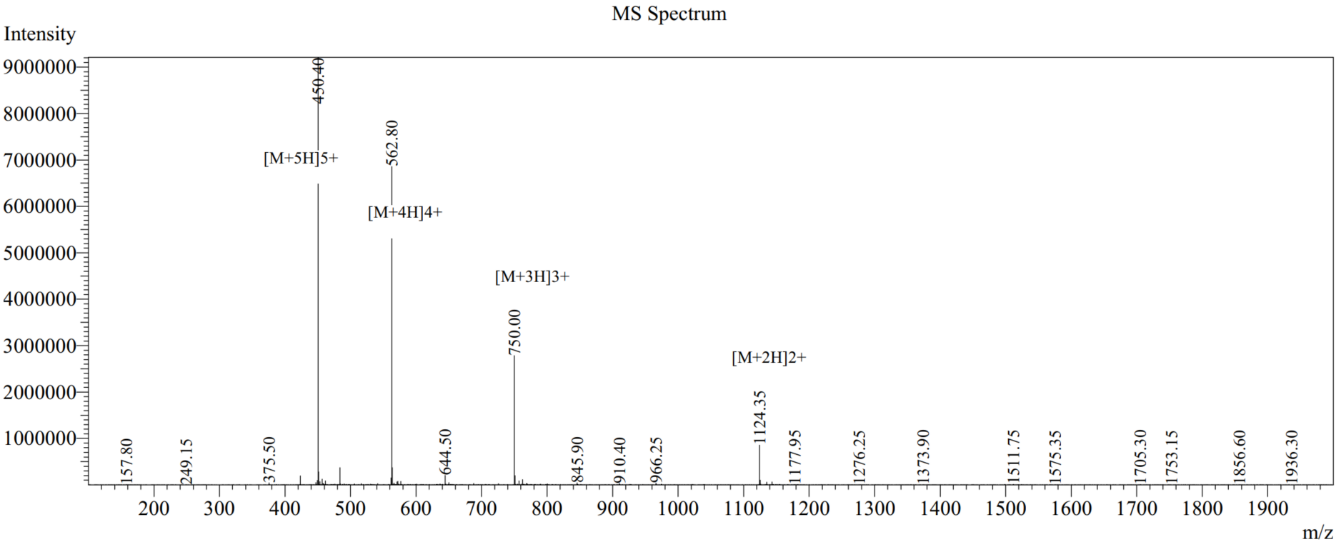


**Figure S15.** ESI-MS of WL-C_8_. Expected mass: 2247.13; Measured average mass 2247.00.
